# High-Performance Thermochromic Multilayer Coatings of W‑Doped VO_2_ Nanoparticles Dispersed in an SiO_2_ Matrix Prepared on Glass at a Low Temperature

**DOI:** 10.1021/acsanm.5c05734

**Published:** 2026-02-13

**Authors:** Jaroslav Vlček, Michal Kaufman, Elnaz Mohammadi Nia, Jiří Houška, Jiechao Jiang, Radomír Čerstvý, Stanislav Haviar, Efstathios I. Meletis

**Affiliations:** † Department of Physics and NTIS − European Centre of Excellence, 48232 University of West Bohemia in Pilsen, Univerzitní 8, 30100 Pilsen, Czech Republic; ‡ Department of Materials Science and Engineering, 12329 The University of Texas at Arlington, Arlington, 76019 Texas, United States

**Keywords:** doped vanadium dioxide, nanoparticles, silicon dioxide matrix, strongly thermochromic coating, low transition temperature, low-temperature synthesis, smart windows

## Abstract

We report a high-performance thermochromic VO_2_-based coating prepared on standard glass at a low substrate temperature of 350 °C without opening the vacuum chamber to the atmosphere. It is formed by four layers of W-doped VO_2_ nanoparticles dispersed in the SiO_2_ matrix. The coating exhibits a transition temperature of 33 °C with an integral luminous transmittance of 65.4% (low-temperature state) and 60.1% (high-temperature state), and a modulation of the solar energy transmittance of 15.3%. Such a combination of properties, together with the low temperature during preparation, fulfills the requirements for large-scale implementation on building glass and has not been reported yet.

## Graphical abstract

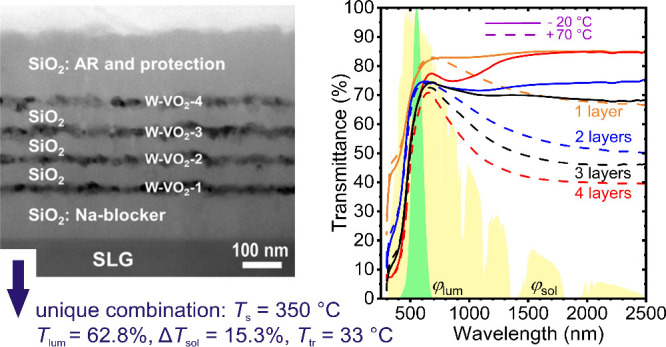

Vanadium dioxide (VO_2_) exhibits a reversible phase transition from a low-temperature monoclinic VO_2_(M1) semiconducting phase to a high-temperature tetragonal VO_2_(R) metallic phase at a transition temperature (*T*_tr_) of ∼68 °C.^1^ *T*_tr_ can be lowered using doping with other elements (such as W).^2–6^ The automatic response to temperature and the abrupt decrease of infrared transmittance with almost unchanged luminous transmittance at the transition into the metallic state make VO_2_-based coatings a promising candidate for thermochromic (TC) smart windows reducing the energy consumption of buildings.

To meet the requirements for large-scale implementation on building glass, VO_2_-based coatings should satisfy the following strict criteria simultaneously (see the review^3^ and refs therein): integral luminous transmittance *T*_lum_ > 60%, modulation of the solar energy transmittance Δ*T*_sol_ > 10%, *T*_tr_ near room temperature, long-term environmental stability, and an appealing color. Moreover, the maximum substrate temperature (*T*_s_) during preparation (deposition and possible postannealing) should be ∼400 °C or lower, as required by industrially important substrates, such as soda-lime glass (SLG), ultrathin flexible glass, or polymer foils. Here, a major challenge is to achieve the high *T*_lum_ and Δ*T*_sol_ at the aforementioned decreased values of *T*_tr_ ^3–8^ and *T*_s_.^3,6–8^ To the best of our knowledge, the simultaneous fulfillment of these requirements has been reported for only two TC coatings up to now (see the comparisons^4–6^ of the state-of-the-art works): a three-layer YSZ/V_0.855_W_0.018_Sr_0.127_O_2_/SiO_2_ coating, where YSZ denotes the yttria-stabilized zirconia, with the average *T*_lum_ = 62.2%, Δ*T*_sol_ = 11.2% and *T*_tr_ = 22 °C, which was prepared on glass at *T*_s_ = 320 °C,^8^ and a composite W-VO_2_@AA/PVP film, where AA and PVP denote l-ascorbic acid and polyvinylpyrrolidone, respectively, with the average *T*_lum_ = 69.3%, Δ*T*_sol_ = 10.2% and *T*_tr_ = 34.5 °C, which was prepared on glass at *T*_s_ = 60 °C.^6^

Recently, valuable results concerning TC VO_2_-based films for future smart-window applications have been achieved.^9–12^ A two-step process consisting of magnetron sputter deposition and postannealing in an oxidizing environment made it possible to produce a discontinuous VO_2_ structure on quartz glass,^9^ quartz^10,12^ and sapphire^11^ substrates. Note that magnetron sputter deposition with its versatility and ease of scaling up to large substrate sizes^13^ is probably the most important preparation technique of TC VO_2_-based coatings.^3^ The layers of the subwavelength (to minimize scattering) VO_2_ nanoparticles with a lower visible-range refractive index (*n*) and extinction coefficient (*k*) than those of the compact VO_2_ layer^9^ exhibited very high *T*_lum_ due to a decreased visible-range reflectance and absorption.^9,12^ At the same time, the localized surface plasmon resonance (LSPR) on the surface of VO_2_(R) metallic nanoparticles enhanced Δ*T*_sol_ significantly due to an increased absorption in the near-infrared region. However, the *T*_tr_ values of these nanostructured materials are too high (53–66 °C), as the fabricated VO_2_ nanoparticles have not been doped, and the *T*_s_ values during the preparation of these materials are too high (450–600 °C) for the aforementioned industrially important substrates.

Here, we present a high-performance (average *T*_lum_ = 62.8% and Δ*T*_sol_ = 15.3%) TC VO_2_-based coating with a decreased *T*_tr_ = 33 °C and enhanced environmental protection, which was prepared on conventional SLG at a low *T*_s_ = 350 °C. It is formed by four layers of W-doped VO_2_ nanoparticles dispersed in a SiO_2_ matrix. We discuss the design and phase composition of the coating, microstructure, elemental composition, and surface morphology of the individual nanocomposite layers, the TC properties of multilayer coatings with one to four nanocomposite layers, and their synthesis performed without opening the vacuum chamber to the atmosphere.

Prior to application of the TC coating, the 1 mm thick SLG substrate was covered without external heating by a 100 nm thick SiO_2_ layer blocking Na diffusion from SLG.^14^ Then, a 10 nm thick V–W film was deposited also without external heating. Subsequently, the coating was annealed up to 350 °C for 1 h (including ∼15 min heating up) in pure O_2_ at *p*_O_2__ = 15 Pa in the same vacuum chamber. After spontaneous cooling below 50 °C, a 50 nm thick SiO_2_ film was deposited. This three-step process was repeated four times. A thicker (160 nm) top SiO_2_ film was applied (Figures 1a, S1) to enhance the antireflection (AR) effect and to provide mechanical and environmental protection. The thickness of 160 nm was a result of the optimization of the *T*_lum_ and Δ*T*_sol_ values. The coatings were produced in an ultrahigh vacuum multimagnetron sputter device (ATC 2200-V AJA International Inc.) equipped by unbalanced magnetrons with planar targets.^8^ The V–W films were deposited by pulsed DC magnetron sputtering of a single V–W (4.0 wt % W corresponding to 1.14 at. % W) target at *p*_Ar_ = 1 Pa using a unipolar pulsed DC power supply (TruPlasma Highpulse 4002 TRUMPF Huettinger). The pulse duration was 50 μs at a frequency of 500 Hz, and the deposition-averaged target power density was 14.9 Wcm^–2^. The SiO_2_ films with *n* of 1.46 and *k* < 10^–3^ at λ = 550 nm were deposited by midfrequency bipolar dual magnetron sputtering of two Si targets at *p*_Ar_ = 1 Pa and *p*_O_2__= 0.2 Pa using a bipolar dual power supply (TruPlasma Bipolar 4010 TRUMPF Huettinger). The pulse duration was 10 μs at a frequency of 50 kHz, and the deposition-averaged target power density was ∼8 W cm^–2^.

**Figure 1:**
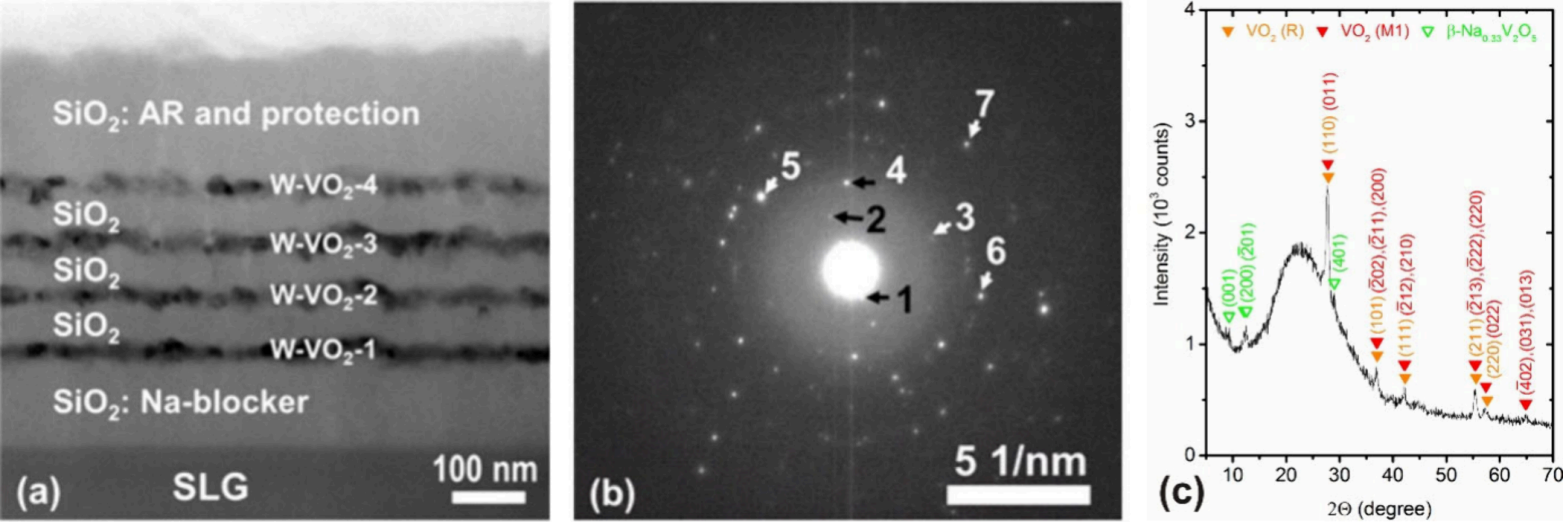
(a) Cross-sectional TEM image of the multilayer coating with four layers of W-doped VO_2_ nanoparticles dispersed in a SiO_2_ matrix on 1 mm thick soda-lime glass. (b) SAED pattern taken from the coating with the *d*-spacings of the diffraction spots 1, 2, 3 (and 4), 5 (and 6) and 7 equal to 9.90 Å, 4.89 Å, 3.24 Å, 2.43 Å and 2.14 Å, respectively. (c) X-ray diffraction pattern taken at *T*_m_ = 25 °C from the coating. The main diffraction peaks of VO_2_(M1), VO_2_(R) and β-Na_0.33_V_2_O_5_ are marked.

The thickness of the as-deposited V–W and SiO_2_ films and the optical constants of the SiO_2_ films were measured by spectroscopic ellipsometry using a J.A. Woollam Co. Inc. VASE instrument. The room-temperature crystal structure was characterized by X-ray diffraction using a PANalytical X’Pert PRO diffractometer working with Cu Kα radiation at a glancing incidence of 1°. The fine microstructure was investigated by transmission electron microscopy (TEM). Selected-area electron diffraction (SAED) patterns and TEM and HRTEM images were recorded in a Hitachi H-9500 electron microscope that is attached with a Gatan SC-1000 Orius CCD camera and an EDAX energy-dispersive spectroscopy (EDS) system. The surface morphology of the nanocomposite layers was investigated by scanning electron microscopy (SEM) using a Hitachi SU-70 microscope. The coating transmittance (*T*) was measured by spectrophotometry using the Agilent CARY 7000 instrument with an in-house made heat/cool cell. The measurements were performed in the wavelength range of 300–2500 nm for *T*_ms_ = −20 °C and *T*_mm_ = 70 °C. The hysteresis of *T* was measured at λ = 2500 nm in the range *T*_m_ = −20 to 70 °C. The aforementioned quantities *T*_lum_ and *T*_sol_ and their modulations are defined as

$$T_{lum}\left(T_{m}\right)=\frac{\int_{380}^{780}\varphi_{lum}\left(\lambda \right)\varphi_{sol}\left(\lambda \right)T\left(T_{m},\lambda \right)d\lambda }{\int_{380}^{780}\varphi_{lum}\left(\lambda \right)\varphi_{sol}\left(\lambda \right)d\lambda }$$

$$\Delta T_{lum}=T_{lum}\left(T_{ms}\right)-T_{lum}\left(T_{mm}\right)$$

$$T_{sol}\left(T_{m}\right)=\frac{\int_{300}^{2500}\varphi_{sol}\left(\lambda \right)T\left(T_{m},\lambda \right)d\lambda }{\int_{300}^{2500}\varphi_{sol}\left(\lambda \right)d\lambda }$$

$$\Delta T_{sol}=T_{sol}\left(T_{ms}\right)-T_{sol}\left(T_{mm}\right)$$

where φ_lum_ is the luminous sensitivity of the human eye and φ_sol_ is the solar irradiance spectrum at an air mass of 1.5.^15^ The average luminous transmittance is defined as *T*_lum_ = [*T*_lum_(*T*_ms_) + *T*_lum_(*T*_mm_)]/2.

Figure 1 presents a cross-sectional TEM image, a SAED pattern, and an XRD pattern taken from the high-performance TC coating on SLG. The coating is composed (Figure 1a) of a bottom ∼100 nm thick SiO_2_ layer, four layers of W-doped VO_2_ nanoparticles (fabricated by annealing the as-deposited V–W films) dispersed in a SiO_2_ matrix, which are separated by three ∼50 nm thick SiO_2_ layers, and a top ∼160 nm thick SiO_2_ layer. The diffraction spots 1 and 2 in Figure 1b probably correspond to the (001) and (002) planes, respectively, of β-Na_0.33_V_2_O_5_ (PDF 01-084-8341),^16^ while the diffraction spot 3 (and 4) corresponds to VO_2_(M1)­(011) planes (PDF 04-003-2035) or to VO_2_(R)­(110) planes (PDF 01-073-2362), the diffraction spot 5 (and 6) corresponds to a superposition of VO_2_(M1)­(2̅02), (2̅11) and (200) planes or to VO_2_(R)­(101) planes, and the diffraction spot 7 corresponds to a superposition of VO_2_(M1)­(2̅12) and (210) planes or to VO_2_(R)­(111) planes.

The crystalline phases identified in the TC coating by XRD (Figure 1c) are in accordance with those identified in Figure 1b. Besides a small contribution of β-Na_0.33_V_2_O_5_ indicating that there was not fully blocked Na diffusion from SLG during not yet fully optimized thermal oxidization, all other peaks are identified as diffraction peaks of either low-temperature VO_2_(M1) or high-temperature VO_2_(R). These TC phases are difficult to distinguish, and they are actually expected to be present simultaneously because the measurement temperature *T*_m_ = 25 °C is close to the transition temperature *T*_tr_ = 33 °C.^8^

Figure 2 presents zoom-in cross-sectional TEM images of the W-VO_2_-4 and W-VO_2_-1 composite layers (Figure 1a), formed by two-dimensional arrays of the W-doped VO_2_ nanoparticles in a SiO_2_ matrix, with the corresponding EDS spectra and HRTEM images of single W-doped VO_2_ nanoparticles. As can be seen in Figures 2b,e, the X-ray EDS analysis confirmed that both layers exhibit the presence of only V, W, Si, O and Na. The *d*-spacing of the lattice fringes related to the W-doped VO_2_ nanoparticle in the W-VO_2_-4 layer (Figure 2c) is about 3.24 Å, corresponding to VO_2_(M1)­(011) planes with *d* = 3.21 Å or to VO_2_(R)­(110) planes with *d* = 3.19 Å. The vertical size of this nanoparticle is ∼42 nm. The *d*-spacing of the lattice fringes related to the W-doped VO_2_ nanoparticle in the W-VO_2_-1 layer (Figure 2f) is about 2.14 Å, corresponding to a superposition of VO_2_(M1)­(2̅12) and (210) planes with *d* = 2.15 and 2.14 Å, respectively, or to VO_2_(R)­(111) planes with *d* = 2.14 Å or even to the orthorhombic VO_2_(P)­(220) planes with *d* = 2.18 Å (PDF 00-025-1003). The vertical size of this nanoparticle is ∼22 nm. Note that the vertical size of some of the nanoparticles is at least 4.2× larger than the V–W film thickness (42 nm compared to 10 nm), while the oxidation of V to VO_2_ increases the volume per metal atom only 2.1× (from ∼14 to ∼29 Å^3^). This represents another fingerprint (in parallel to EDS) of the driving force toward dewetting. However, as can be seen in Figures 2a,d, it is very probable that at least some of the W-doped VO_2_ nanoparticles are connected to each other in the W-VO_2_ layers.^9^

**Figure 2:**
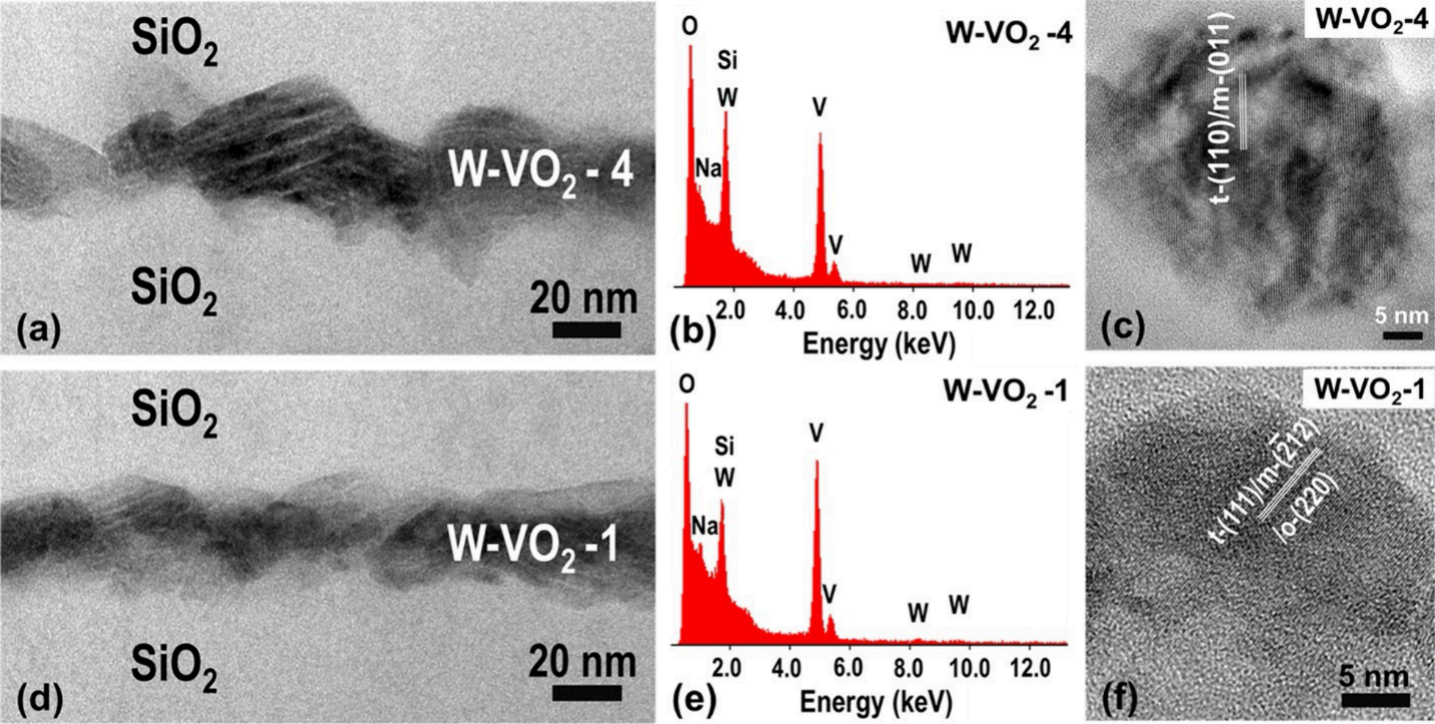
(a and d) Zoom-in cross-sectional TEM images and (b and e) EDS spectra of the W-VO_2_-4 layer and the W-VO_2_-1 layer, respectively. (c and f) HRTEM images of W-doped VO_2_ nanoparticles from the same layers.

Figure 3 presents top-view SEM images of the first layer of W-doped VO_2_ nanoparticles fabricated on the Na-blocking SiO_2_ layer. The diameter of most particles is 41–55 nm, while the spacing between them is 7–21 nm. Moreover, elongated particles with a length up to 150–400 nm or even 1 μm were identified. The surface morphology of this layer is different from the layers with well-separated, uniformly distributed VO_2_ nanoparticles of homogeneous size ≤ 200 nm, which were fabricated by annealing the as-deposited V films in a mixture of Ar and O_2_ at *T*_s_ ≥ 500 °C.^10,12^ Recently, valuable results explaining the dewetting process in undoped and W-doped VO_2_ films fabricated by annealing the corresponding as-deposited films in pure O_2_ have been reported.^17^ It has been shown that a higher temperature (≥600 °C) and a longer annealing time are required for the dewetting of W-doped VO_2_ nanoparticles.

**Figure 3:**
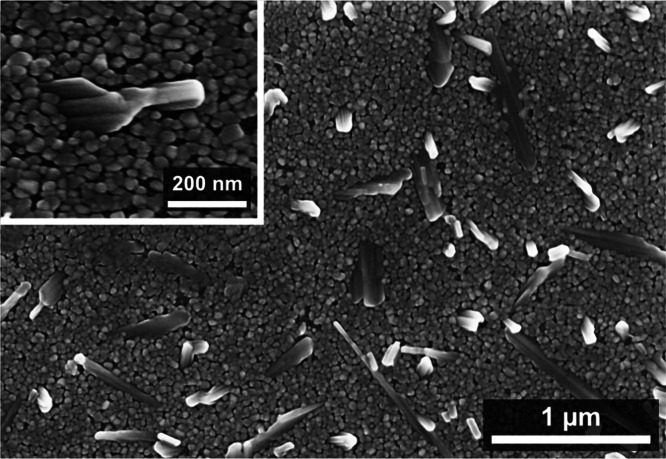
SEM images (top view) of the first layer of W-doped VO_2_ nanoparticles fabricated without any SiO_2_ overlayer on the SiO_2_ layer blocking Na diffusion from the glass substrate (see Figure 1a).

Figure 4 and Table 1 quantify that the presented industry-friendly low-temperature methodology allowed us to prepare strongly TC coatings (large modulation in the infrared in Figure 4a) with a lowered transition temperature (*T*_tr_ of at most 33 °C in Figure 4b). Taking into account the same composition of the V–W target and the metal sublattice of the produced W-doped VO_2_ nanoparticles, the *T*_tr_ gradient of at least −24 °C per atom % of W is larger than most gradients reported previously.^3^ Here, we assumed *T*_tr_ ∼ 60 °C of thin-film VO_2_;^3^ *T*_tr_ = 68 °C of bulk VO_2_ would lead to an even larger gradient. This indicates the successful incorporation of W into the metal sublattice of VO_2_.

**Figure 4:**
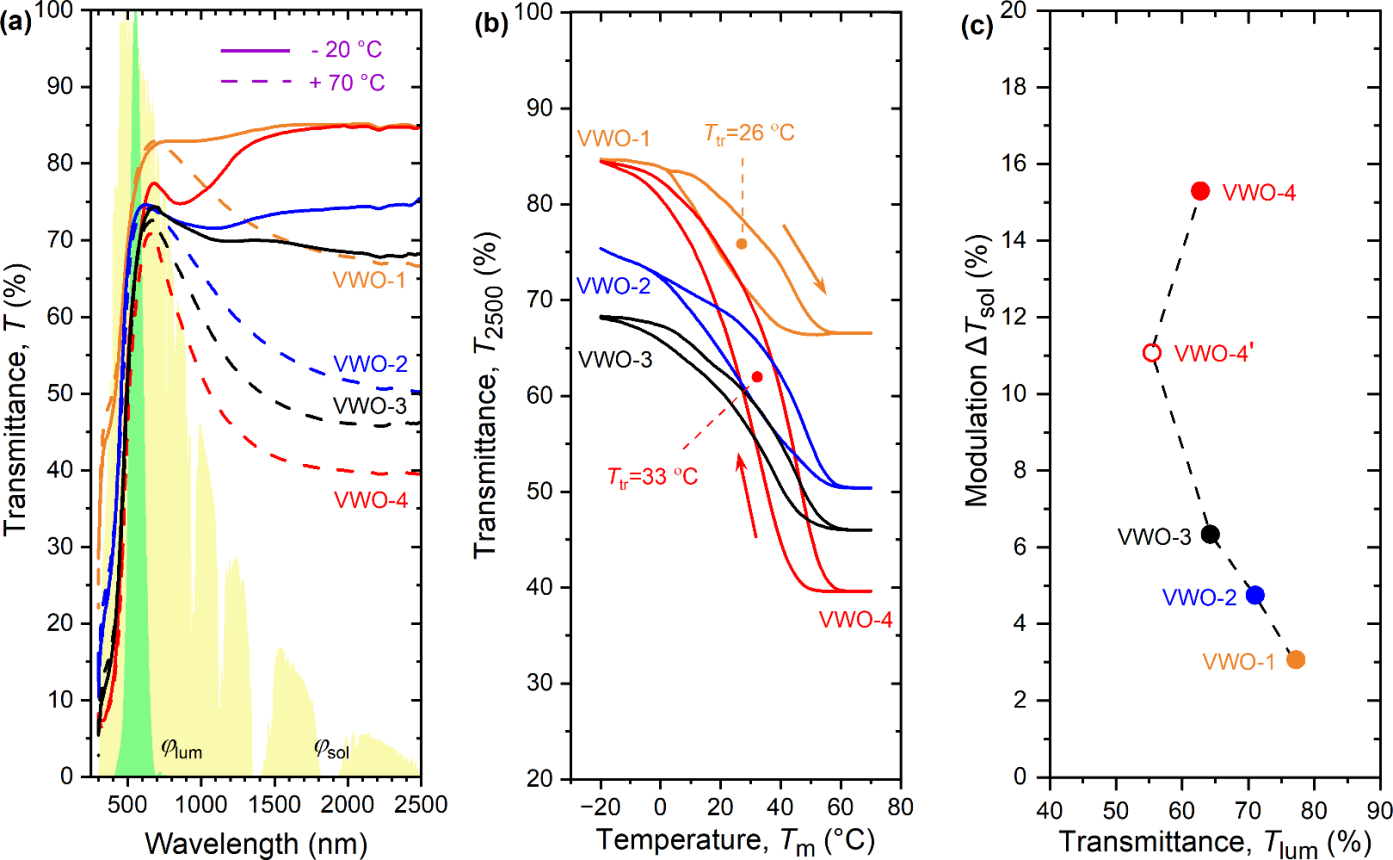
(a) Spectral transmittance measured at *T*_ms_ = −20 °C and *T*_mm_ = 70 °C for four coatings with one (VWO-1), two (VWO-2), three (VWO-3) and four (VWO-4) layers of W-doped VO_2_ nanoparticles with approximately 50 nm thick SiO_2_ overlayers, except for the VWO-4 coating with an approximately 160 nm thick SiO_2_ overlayer, which were fabricated successively on the SiO_2_ layer blocking Na diffusion from the glass substrate (see Figure 1a). The contours of the shaded areas represent the luminous sensitivity of the human eye (φ_lum_) and the solar irradiance spectrum (φ_sol_), normalized to maxima of 100%. (b) Temperature dependences of the transmittance at 2500 nm for the same coatings. The transition temperatures are given for the VWO-1 and VWO-4 coatings. (c) Average integral luminous transmittance and the modulation of the solar energy transmittance for the same coatings and for the VWO-4′ coating with an approximately 50 nm thick SiO_2_ overlayer.

**Table 1:** Integral Luminous and Solar Energy Transmittance [*T*_lum_(*T*_m_) and *T*_sol_(*T*_m_), respectively] Measured at *T*_ms_ = −20 °C and *T*_mm_ = 70 °C, Together with the Corresponding Modulations Δ*T*_lum_ and Δ*T*_sol_, and the Transition Temperature, *T*_tr_, Corresponding to the Middle of Hysteresis Curves (Figure 4b), for Five Coatings with One (VWO-1), Two (VWO-2), Three (VWO-3) and Four (VWO-4′ and VWO-4) Layers of W-Doped VO_2_ Nanoparticles with Approximately 50 nm Thick SiO_2_ Overlayers, Except for the VWO-4 Coating with Approximately 160 nm Thick SiO_2_ Overlayer, Which Were Fabricated Successively on SiO_2_ Layer Blocking Na Diffusion from the Soda-Lime Glass Substrate (Figure 1a)

Coating	*T*_lum_(*T*_ms_) (%)	*T*_lum_(*T*_mm_) (%)	Δ*T*_lum_ (%)	*T*_sol_(*T*_ms_) (%)	*T*_sol_(*T*_mm_) (%)	Δ*T*_sol_ (%)	*T*_tr_
VWO-1	76.3	77.1	–0.8	76.8	73.7	3.1	26
VWO-2	71.9	70.5	1.4	67.2	62.5	4.7	31
VWO-3	64.9	63.9	1.0	62.4	56.1	6.3	32
VWO-4′	56.8	53.8	3.0	55.8	44.7	11.1	33
VWO-4	65.4	60.1	5.3	66.1	50.8	15.3	33

The gradual increase of the number of TC layers from 1 to 4 leads to monotonically but relatively slowly decreasing *T*_lum_, from 76.3 to 77.1% through 70.5–71.9% and 63.9–64.9% to 53.8–56.8%. While these values are already affected by the lower *n* and in turn lower reflectance of SiO_2_-containing composite TC layers compared to compact homogeneous W-doped VO_2_, altering the AR effect by the transition from 50 to 160 nm SiO_2_ overlayer leads to a further enhancement of *T*_lum_ from 53.8–56.8% to 60.1–65.4%. This has to be interpreted in the context given by the total thickness of the four V–W layers of ∼40 nm, corresponding to ∼84 nm of W-doped VO_2_ with the same areal density of metal atoms. The present *T*_lum_ = 60.1–65.4% of the coating based on doped VO_2_ nanoparticles is actually higher than the *T*_lum_ of the coatings^3,8^ based on compact pure or doped VO_2_ layers having thicknesses well below 84 nm, and dramatically higher than the *T*_lum_ predicted^3,8^ for coatings based on compact layers having a similar thickness of 80 nm. Moreover, there is a potential for further *T*_lum_ enhancement by fine-tuning of *n* and the thickness of the AR overlayer, while the aforementioned predictions assumed an optimized one. One reason for this success, which increases the potential to achieve high Δ*T*_sol_ at sufficient *T*_lum_, is the nonlinear dependence of *k* of the composite TC layer on its composition. To give an example, assuming a SiO_2_ matrix with spherical inclusions of W-doped VO_2_, the Maxwell-Garnett effective medium approximation at equal volume fractions of both these phases yields *k* at λ = 550 nm of only ∼63% of the corresponding arithmetic mean.

In parallel to the evolution of *T*_lum_, the gradual increase of the number of TC layers from 1 to 4 leads to increasing Δ*T*_sol_ from 3.1% through 4.7% and 6.3% to 11.1%, and the transition from 50 to 160 nm SiO_2_ overlayer leads to its further enhancement to 15.3%. While the basic factor is the increasing transmittance modulation in the infrared with increasing amount of the TC phase, there are three more phenomena to note. First, there is almost monotonically increasing transmittance modulation also in the visible, from Δ*T*_lum_ = −0.8% to 5.3% (multiplied by high φ_sol_ and in turn responsible for about a quarter of the Δ*T*_sol_ enhancement). Second, Figure 4a shows that the 160 nm thick SiO_2_ overlayer leads to exceptionally nonmonotonic low-temperature *T*(λ) with a (second-order) maximum in the visible complemented by a (first-order) maximum in the infrared, increasing the contribution of infrared wavelengths to Δ*T*_sol_. Third, the absorption in the high-temperature state, and in turn Δ*T*_sol_, is arguably enhanced by LSPR at the boundary between metallic W-doped VO_2_(R) nanoparticles and dielectric SiO_2_. The resonance condition is prone to be close to fulfilled in the near-infrared.^2,3^ This explains that the coating performance at a given areal density of metal atoms is better than that of coatings based on compact pure or doped VO_2_ layers (including coatings which utilize the previous two phenomena)^3,8^ not only in terms of *T*_lum_ (previous paragraph) but also in terms of Δ*T*_sol_. As some of the nanoparticles are presently interconnected, the potential of this phenomenon may be even larger. Such nonidealities may be behind different high-temperature *T*(λ) of different VO_2_-based coatings utilizing LSPR, sometimes^10,12^ exhibiting a minimum in the near-infrared, sometimes^11^ (consistently with our result) monotonically decreasing in the near-infrared. Collectively, the presented coating characteristics show that while the well-known^2,3^ trade-off between *T*_lum_ and Δ*T*_sol_ due to varied amounts of the TC material takes place also in the present case, the specific pairs of these values (Figure 4c) are beyond anything reported until now at such a low *T*_tr_.

In summary, we present a high-performance (average *T*_lum_ = 62.8% and Δ*T*_sol_ = 15.3%) TC VO_2_-based coating with a decreased *T*_tr_ = 33 °C and enhanced environmental protection, which was prepared on conventional glass at a low *T*_s_ = 350 °C without opening the vacuum chamber to the atmosphere. It is formed by four layers of W-doped VO_2_ nanoparticles dispersed in a SiO_2_ matrix. Such a combination of properties, together with the low temperature during a three-step preparation process (magnetron sputter depositions and postannealing in oxygen), fulfills the requirements for large-scale implementation on building glass and has not been reported yet. Necessary reduction in the number of layers and shortening the preparation process would move us closer to reducing the energy consumption of buildings by applying this kind of coating on windows and glass facades.

## Supplementary Material



## Notes

Schematic illustration of the thermochromic coating. Basic characteristics of the W-doped VO_2_ nanoparticles. Fabrication method used for the preparation of the W-doped VO_2_ nanoparticles. (PDF)
