# Supplementary material for: High-Performance Thermochromic Multilayer Coatings of W‑Doped VO2 Nanoparticles Dispersed in an SiO2 Matrix Prepared on Glass at a Low Temperature
Source: ACS Appl Nano Mater. 2026 Feb 13;9(8):3597–602. doi: 10.1021/acsanm.5c05734 (PMC12954752; doi:10.1021/acsanm.5c05734)
Supplement: Supplementary file 1 [file an5c05734_si_001.pdf]

Supporting Information

High-Performance Thermochromic Multilayer Coatings of W-Doped VO_2 Nanoparticles Dispersed in SiO_2 Matrix Prepared on Glass at a Low Temperature

Jaroslav Vlček,[a,*] Michal Kaufman,[a] Elnaz Mohammadi Nia,[a] Jiří Houška,[a] Jiechao Jiang,[b] Radomír Čerstvý,[a] Stanislav Haviar,[a] Efstathios I. Meletis [b]

E-mail address: vlcek@kfy.zcu.cz (J. Vlček)

[a] Department of Physics and NTIS – European Centre of Excellence, University of West Bohemia in Pilsen, Univerzitní 8, 30100 Pilsen, Czech Republic

[b] Department of Materials Science and Engineering, The University of Texas at Arlington, Arlington, 76019, TX, USA

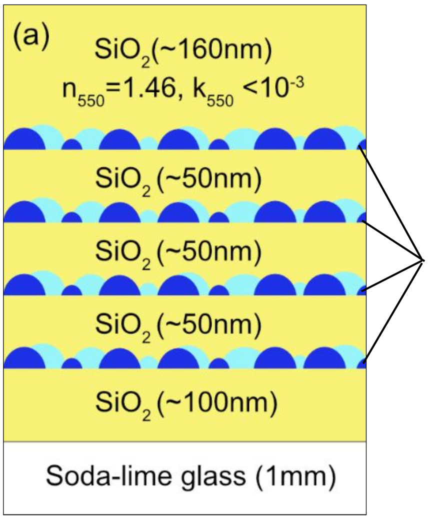

(b)

Two-dimensional arrays of W-VO_2 nanoparticles: H=20-45nm, D=41-55nm and S=7-21nm

↔

(c)

Solid-state dewetting from 10nm V-W films at 350 °C in O_2: $V_{0.989}W_{0.011}+O_2 \rightarrow V_{0.989}W_{0.011}O_2$

Figure S1. (a) Schematic illustration of the thermochromic coating. (b) Basic characteristics of the W-doped VO_2 nanoparticles where H, D and S are their height and diameter, and spacing between them, respectively. (c) Fabrication method used for the preparation of the W-doped VO_2 nanoparticles.
